# What aspects of intentional rounding work in hospital wards, for whom and in what circumstances? A realist evaluation protocol

**DOI:** 10.1136/bmjopen-2016-014776

**Published:** 2017-01-09

**Authors:** Ruth Harris, Sarah Sims, Ros Levenson, Stephen Gourlay, Fiona Ross CBE, Nigel Davies, Sally Brearley, Giampiero Favato, Robert Grant

**Affiliations:** 1 Florence Nightingale Faculty of Nursing and Midwifery, King's College London, London, UK; 2 Independent Consultant, London, UK; 3 Kingston Business School, Kingston University, Kingston-Upon-Thames, London, UK; 4 Faculty of Health, Social Care and Education, Kingston University and St George's, University of London, London, UK; 5 Faculty of Health and Social Sciences, University of Bedfordshire, Luton, UK; 6 Institute of Leadership and Management in Health (ILMH), Kingston University, Kingston-Upon-Thames, London, UK

## Abstract

**Introduction:**

Intentional rounding (IR) is a structured process whereby nurses in hospitals carry out regular checks, usually hourly, with individual patients using a standardised protocol to address issues of positioning, pain, personal needs and placement of items. The widespread implementation of IR across the UK has been driven by the recommendations of the Francis Inquiry although empirical evidence of its effectiveness is poor. This paper presents a protocol of a multimethod study using a realist evaluation approach to investigate the impact and effectiveness of IR in hospital wards on the organisation, delivery and experience of care from the perspective of patients, their family members and staff.

**Methods and analysis:**

The study will be conducted in four phases. Phase 1: theory development using realist synthesis to generate hypotheses about what the mechanisms of IR may be, what particular groups may benefit most or least and what contextual factors might be important to its success or failure which will be tested in subsequent phases of the study. Phase 2: a national survey of all NHS acute trusts to explore how IR is implemented and supported across England. Phase 3: case studies to explore how IR is implemented ‘on the ground’, including individual interviews with patients, family members and staff, non-participant observation, retrieval of routinely collected patient outcomes and cost analysis. Phase 4: accumulative data analysis across the phases to scrutinise data for patterns of congruence and discordance and develop an overall evaluation of what aspects of IR work, for whom and in what circumstances.

**Ethics and dissemination:**

The study has been approved by NHS South East Coast—Surrey Research Ethics Committee. Findings will be published in a wide range of outputs targeted at key audiences, including patient and carer organisations, nursing staff and healthcare managers.

**Strengths and limitations of this study**

- The study will clearly articulate the preliminary theories and assumptions about intentional rounding (IR) and how it is expected to work.
- The study will test and refine these theories throughout the study using existing empirical evidence, a national survey to investigate implementation and local case studies.
- The study design allows these theories to be examined in different acute care delivery contexts allowing established assumptions about IR and the outcomes of IR to be examined.
- Applying a realist evaluation approach can be challenging, and there may be limited evidence to support some elements of the programme theory.

## Introduction

**Figure 1:**
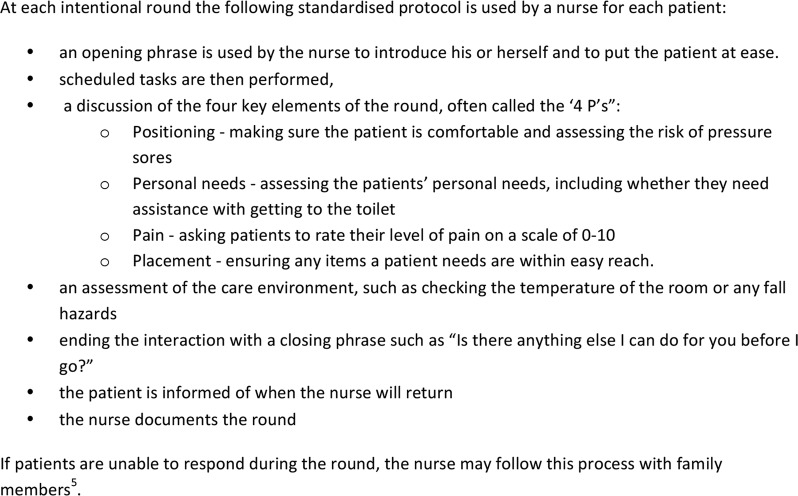
Typical IR schedule in acute ward settings.^5–7^

‘Patients first and foremost’^1^ is the priority for the NHS. However, as demand for health services is increasing so are concerns that the delivery of patient care is lacking in compassion and less tailored to individual patient need, particularly for older people.^2^ These concerns were highlighted in the Francis Inquiry,^3^ which examined evidence about the reasons for the failures in patient care at Mid Staffordshire NHS Trust and made key recommendations to strengthen local systems to deliver safe, compassionate, patient-centred care. Engagement with patients is highlighted as a mechanism to promote well-being and improve patients' experience of healthcare treatment and this is seen as principally the role of nursing staff (Vol III, p1606). One of the Inquiry's recommendations states that ‘Regular interaction and engagement between nurses and patients and those close to them should be systematised though regular ward rounds’ (Vol III, p1610) and refers to the use of a regular ward round as suggested by the Prime Minister in January 2012. Following this announcement, the majority of NHS trusts have introduced intentional rounding (IR), a structured process whereby nurses in hospitals carry out regular checks, usually hourly, with individual patients using a standardised protocol to address issues of positioning, pain, personal needs and placement of items. Conducting hourly rounds is not a new nursing concept, and ‘care rounds’ or ‘comfort rounds’ have been carried out for many years by nurses.^4^ ^5^ However, IR offers a more structured version of this process, using a standardised protocol purposively aimed at keeping patients comfortable and safe (see figure 1 for typical IR protocol).

Evidence in the form of local audits and published studies has highlighted numerous benefits of IR, including a reduction in call bell use, falls and pressure sores as well as increased patient satisfaction and the delivery of care that demonstrates compassion.^4^ ^7–9^ However, there is limited research to support this and most of this has been conducted in US hospitals, therefore findings may not be applicable to other international healthcare contexts. Substantial limitations to the evidence base for IR have also recently been highlighted by Snelling,^10^ who states that results asserting the benefits of IR should be interpreted with caution due to concerns around selection bias, potential conflict of interest, study design and data analysis. Other reviews have also highlighted weaknesses in the design of IR studies.^5^ ^11^

Little is also known about how NHS healthcare trusts in the UK currently define IR, whether there is consistency in its implementation or whether it has had any unintended consequences on other aspects of nursing activity. These key issues have been highlighted as requiring further investigation.^5^ ^10^ ^11^ Thus, while IR is intuitively a good idea and implemented in a majority of NHS hospital trusts, there is currently no robust research evidence to support its widespread adoption in the UK and internationally. With the increased scrutiny as a result of the Francis Inquiry and financial pressures on the NHS, it is important to establish evidence of the effectiveness and costs of IR by finding out what works (or otherwise), for whom and in what circumstances.

## Aims and objectives

This study aims to investigate the impact and effectiveness of IR in hospital wards on the organisation, delivery and experience of care from the perspective of patients, their family members and staff. The research question is: ‘What is it about IR in hospital wards that works, for whom and in what circumstances?’ We will investigate this at the three levels of the organisation and delivery of health services: national, service provider organisation and individual ward/unit. We will identify the ways in which the context (ie, the environment and organisation) at each of these levels influences the mechanisms (ie, the assumptions and theories about the ways in which IR achieves its objectives) and the outcomes or impact. The study started in September 2014. However, it has been delayed due to unforeseen circumstances and now will be completed in March 2018. The study objectives are to:

1. Determine how many NHS trusts in England have implemented IR and analyse how this has been developed and supported.
2. Identify how IR has been implemented ‘on the ground’ and evaluate its contribution to the delivery of patient care as a whole and how it fits in alongside other approaches to improving quality and safety.
3. Explore nursing staff, healthcare assistants and other clinical and management staff experiences of IR and how it affects the way they deliver care.
4. Explore patients' and their family members' experiences and perceptions of how IR influences their experiences of care.
5. Investigate the possibility of identifying trends in patient outcomes (retrieved from routinely collected NHS ward data) within the context of the introduction of IR and other care improvement initiatives that have been introduced by using statistical process controls methods such as CUSUM charts.
6. Examine the barriers and facilitators to the successful implementation of IR.
7. Conduct a bottom-up analysis of the costs of IR by identifying the resources used by case study wards to develop and implement it.
8. Synthesise the data from each of the study phases to identify which aspects work, for whom and in what circumstances.

## Project methodology

### Study design and conceptual basis

**Figure 2:**
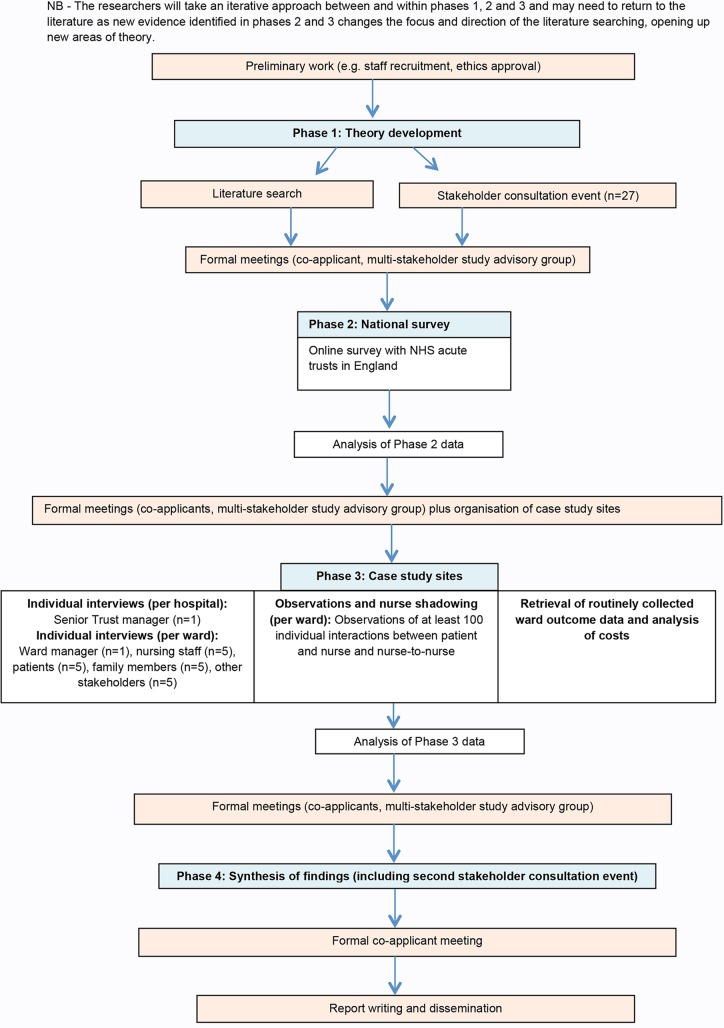
Flow diagram.

A multimethod study design will be undertaken drawing on a realist evaluation approach^12^ (see figure 2). A randomised, experimental study design is not possible as the implementation of IR has been strongly advocated and promoted by the UK government and very few trusts are reported not to have implemented it. Realist evaluation is a theory-driven approach designed for evaluating complex social interventions such as IR,^13^ where the outcomes of an intervention are influenced by the way it is delivered and the context in which it is delivered.^14^ ^15^ It does not seek to answer the question ‘does this intervention work?’ but instead acknowledges that complex social interventions only ever work for certain people, in particular circumstances. The key task of a realist evaluation is to understand and explain these patterns of success and failure by asking the exploratory question: what is it about this intervention that works, for whom and in what circumstances?^12^ ^15^ It achieves this through the identification of context–mechanism–outcome configurations.

The study will be conducted in four phases: (1) theory development; (2) national survey of all NHS acute trusts in England; (3) individual interviews with healthcare staff, patients and their family members, observations of IR and nurse shadowing, retrieval of routinely collected ward outcome data and analysis of costs and (4) synthesis of study findings. The study will be guided throughout its duration by a multistakeholder advisory group consisting of nine NHS senior managers and healthcare professionals and nine patient and carer representatives.

### Phase 1: theory development

As with all social interventions, it can be assumed that IR will work for different stakeholders in various settings in different ways. However, available theory on its potential is limited. Therefore, we will begin with a period of theory development drawing on principles of realist synthesis^16^ to generate hypotheses on what the mechanisms may be, what particular groups may benefit most or least and the contextual factors that might be important to its success or failure. These hypotheses will be interrogated and tested in phases 2 and 3 of the study.

Literature will be identified from electronic searches of databases, including MEDLINE, BMJ Journals, CINAHL, Embase, Internurse, RCN Archive, PsychINFO, HMIC and the Cochrane Library. Reference lists of relevant papers will be scanned and citation searches conducted. Expert advice about generating relevant search terms will be sought from Information Sciences Specialists and revised as additional key words are generated. Grey literature relating to policy and organisational-based material will be sought by searching government and other specialist websites. Papers and other information that satisfy any of the following criteria will be identified as potentially relevant and will be retrieved for review:

- describe or evaluate IR,
- detail its implementation or development in various settings,
- address the experience of individual team members, team leaders, policymakers, patients or their family members around implementing, conducting or experiencing intentional rounds,
- describe the organisational or political context of IR,
- reviews of IR.

Only English language documents will be included. In line with realist methodology, we will not have specific predetermined inclusion and exclusion criteria based on research method or quality, but we will report areas of general weakness in evidence and individual study weakness where appropriate. The abstracts of all papers identified by searches will be screened for suitability. All potentially relevant papers will be retrieved and assessed by a member of the research team using a structured data extraction form. The following information will be recorded for each potentially relevant paper:

- literature item details (type of item, eg, descriptive, evaluative, review),
- area in which the intentional round is situated (eg, acute care, care of older people),
- details of the intentional round (eg, frequency, duration, who it is conducted by),
- outcomes (eg, reduction in call bell use, falls and pressure sores),
- enablers and inhibitors (eg, factors recorded as enabling or inhibiting the implementation or delivery of intentional rounds).

Each data extraction form will be independently examined by at least two members of the research team for inclusion. Data or information from each of the studies selected will be analysed thematically to provide a comprehensive description of the purported mechanisms of IR. Contexts that appear to trigger or inhibit the mechanisms will be identified and outcomes for patients and their family members, healthcare staff, teams and organisations when the mechanism is present or absent will be noted.

In addition to the literature review a stakeholder consultation event will be held, in which key figures associated with IR (eg, Directors of Nursing of NHS hospitals, healthcare staff) plus the study's advisory group will be asked to elicit realist theories on the mechanisms. This process is recommended in realist evaluation, as understanding what key stakeholders know about an intervention and their reasoning for or against its implementation is essential to understanding it. Data from the literature review and the stakeholder consultation event will be synthesised to provide a rich and detailed picture of the intervention of IR.

### Phase 2: national survey of non-specialist NHS acute trusts in England

Phase 2 will explore how IR is currently being implemented and supported nationally within NHS acute trusts across England and the way in which organisational context has influenced its implementation. The findings will inform the in-depth case studies conducted in phase 3, including case study site selection.

**Figure 3:**
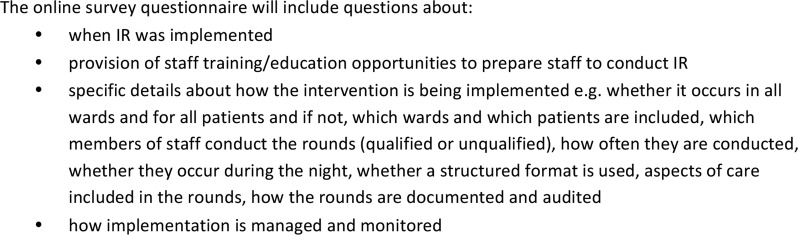
Examples of survey questions to be included in the national survey.

A national survey of all NHS acute trusts in England will be undertaken using an online structured questionnaire (see figure 3 for examples of survey questions) administered to a senior trust manager with responsibility for implementing nursing services. We were advised by local trust nursing directors and managers that this would be the best approach to maximise the response rate. Each trust's Director of Nursing will be contacted directly and asked to complete the survey or forward to a colleague who would be able to complete it. Up to three email reminders will be sent and a clear audit trail will be maintained. Reponses to the survey will be entered into STATA, collated and subjected to quantitative analyses to explore and provide a detailed picture of how IR has been implemented nationally.

### Phase 3: In-depth case studies

Phase 3 will explore the extent to which the concepts of IR identified in phase 1 are compatible with or relevant to modern health service delivery and the experiences of healthcare staff, patients and their family members.

#### Case study settings

Three geographically spread hospitals in England will be purposively selected based on the findings of the national survey in phase 2 to identify sites where IR has been implemented differently (ie, maturity of intervention, structure of process). Within each of these case study locations, the following data will be collected from two wards (one acute, one care of older people):

#### Individual interviews

Within each hospital, individual semistructured interviews will be conducted with a senior trust manager with responsibility for nursing to provide detailed information about the implementation of IR within the trust, including why IR was implemented, staff training needs to conduct rounds and how these were addressed, future development needs and the implementation of other nursing innovations to improve the quality of nurse/patient interactions. On each ward, individual qualitative interviews will be conducted with the ward manager (n=1), ward nursing staff and healthcare assistants (n=5), patients (n=5), family members (n=5) and other stakeholders, for example, doctors, non-nursing managers and therapy staff (n=5). Interviewees will be purposively sampled to attain a range of genders and ethnicities. Healthcare staff will also be purposively sampled to attain a range of professions and grades. Informed consent will be gained from all participants and all interviews will be audio-recorded unless the interviewee requests otherwise.

**Figure 4:**
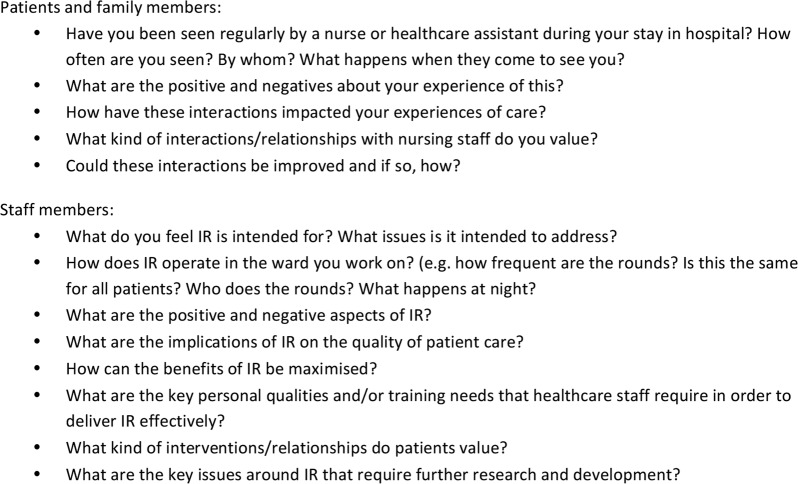
Anticipated questions to be included in interview schedules for patients, family members and staff.

Interview schedules will be informed by the findings from phase 1 and specifically designed to elicit detailed reflections on how the different mechanisms and contexts of IR influence the interviewee and others around them. The findings from phase 1 will be discussed with participants who will be asked how they relate, if at all, to their experience. If the findings are not considered relevant, the reasons for this will be explored. Anticipated key questions for healthcare staff and managers are detailed in figure 4.

It is anticipated that patients and their family members may not be explicitly aware of the term ‘intentional rounding’ and if this is the case the term will not be directly used. Where the interviewee is unaware of the term, the interviewer will instead talk about ‘hourly nursing rounds’ or ask about the regular contact that the patient or their family member has with nursing staff. Anticipated key questions for patients and their family members are detailed in figure 4.

It is anticipated that each interview will last up to an hour. Individual interviews will be transcribed and analysed using framework analysis^17^ to identify themes within the data and to facilitate comparison between case studies.

#### Non-participant observation and nurse shadowing

Non-participant observation of direct patient care will be carried out on each ward over a period of 2–3 weeks to observe how IR is implemented ‘on the ground’. For each case study site, researchers will produce a detailed description of the ward environment, including nursing shift patterns, staffing information, sickness levels and vacancy rates: factors related to ward ecology, including the layout of the ward and screening of beds; and factors related to how IR was implemented, including how the change in practice was initially introduced, staff preparation and training and ongoing development and sustainability. Observational methods will include ‘shadowing’ nursing staff to explore how they interact with patients and each other in relation to IR. Researchers will observe nursing handovers and describe how decisions are made over who conducts intentional rounds (grade of staff, permanent or temporary staff etc), whether the same person conducts the rounds consistently throughout the course of a shift, whether rounds are conducted as they are intended, how they are recorded and what happens afterwards. Researchers will also observe individual interactions between the patient and nurse during a series of intentional rounds. Quality Patient Care Scales (QUALPACS) will also be completed for five patients in each ward. QUALPACS^18^ is an established instrument for assessing the quality of care a patient receives from a nurse using 68-items across the following areas of care: physical, general, psychosocial, communication and professional implications. The instrument is patient-focused, with observations based on who attends the patient's bedside to provide care and how frequently.^19^ The observer watches the care received by selected patients over a 2-hour period and rates each aspect of this care on a scale of 1 (poorest care) to 5 (best care).^19^ ^20^ Researchers will observe at least 100 individual IR interactions between the patient and nurse and between nurses on each ward using these various methods. In all observations, the researcher will record the duration of each individual interaction, how often interactions occur, what patients are asked during their interaction and what care is provided. External factors that impact on the delivery of IR will also be recorded. This will enable us to establish how the intervention fits within the whole nurse experience of the delivery of care and the whole patient experience of receiving care. Observation data will be analysed using descriptive statistics and thematic analysis of ethnographic field notes to identify and describe the mechanisms of IR. The contexts and, where possible, the outcomes associated with the mechanisms will also be identified.

#### Retrieval of routinely collected outcome data

Routinely collected outcome data from the NHS Safety Thermometer will be retrieved for each of the case study wards. The NHS Safety Thermometer is ‘a local improvement tool for measuring, monitoring and analysing patient harms and “harm free” care’ (http://www.hscic.gov.uk/thermometer)**.** Data collected from the NHS Safety Thermometer are available online subject to appropriate permissions but can also be retrieved from the specific case study sites. Quantitative analyses of the NHS Safety Thermometer data will be exploratory and, as per the realist approach, will be specifically tailored to the individual circumstances associated with the implementation of IR on each ward. For example, if IR was introduced on a ward on a set date and had operated without issue ever since, the analyses could compare the NHS Safety Thermometer data from 6 months prior to the introduction of IR to 6 months after its implementation. If, however, IR was introduced on a ward, then terminated for a period of time before starting again, analyses of the NHS Safety Thermometer data could be conducted on a month-by-month basis to explore whether there were any differences in outcomes during these periods. The aim was not to attribute cause and effect but to investigate the possibility of identifying trends in patient outcomes within the context of the introduction of IR and other care improvement initiatives that have been introduced. Statistical process controls methods such as CUSUM charts will be used.

#### Analysis of costs

An exploratory analysis of the costs of IR will be undertaken. The pattern and consistency of resource use to undertake IR will be assessed on each ward as there may be day-to-day variation in completion of IR and grade of staff conducting IR as well as variation between case study wards. A bottom-up approach to costing IR activity will be employed using data collected in the staff interviews, non-participant observation and shadowing and detailed information about ward context. Resource use data will include:

- Duration of IR—time with individual patients and time for the ward as a whole,
- Grade of staff involved in direct contact with patients during IR and in the day-to-day organisation of IR,
- Costs of consumables used in IR, for example, documentation, time sheets/clocks,
- Costs to set up IR, for example, time spent by staff to develop operational guidelines and to change practice,
- Training costs for staff team at initial set up and ongoing training and development needs.

The overall costs of the development and ongoing implementation on a daily basis will be estimated for each case study ward. This will enable comparison of the costs of different approaches to IR development and implementation and provide details of how case study sites differ. The possibility of assessing the costs for the proposed mechanisms generated in the realist synthesis and the contexts within which they are situated will be explored.

Data from the case studies will be subject to within-case and across-case analysis. The six case studies will provide an in-depth realist evaluation of the various contexts, mechanisms and outcomes of IR and will increase understanding of when, how and for whom it has most effect. The researchers will take an iterative approach between and within phases 1, 2 and 3 and may need to return to the literature as new evidence identified in phases 2 and 3 changes the focus and direction of the literature searching, opening up new areas of theory.

### Phase 4: synthesis of findings

Phase 4 involves the accumulative data analysis from phases 1, 2 and 3. Using the realist evaluation framework, the patterns of outcomes produced by IR will be mapped and the researchers will explore whether the hypothesised contexts and mechanisms adequately explained these patterns. Each phase of this study generates data giving different perspectives of IR and these data will be scrutinised for patterns of congruence and discordance to develop an overall evaluation of what aspects of IR work, for whom and in what circumstances. As part of the synthesis process, attendees from the stakeholder consultation event held in phase 1 will be invited to interrogate the findings and consider how they fit with their own knowledge and experience of IR. The synthesised study findings will establish the potential outcomes of the intervention, identify the underlying mechanisms which explain how it produces these effects and highlight the key contextual factors that affect its success or failure. Recommendations can then be made as to how trusts can best target or develop the intervention for particular groups in various settings.

### Dissemination

This study does not alter clinical care and so there are no potential adverse effects. All participants will be informed that they are free to refuse to participate or withdraw from the study at any time.

The team will disseminate the findings to a range of stakeholders within a planned programme. We will draw on the networks and expertise of the study advisory group and collaborators to disseminate the research outputs widely and appropriately. Key audiences include patient and carer organisations, clinical nursing staff, nursing managers and directors of nursing who have responsibility for the provision of nursing care, managers and directors within healthcare organisations with responsibility to provide high-quality services within budget and healthcare policymakers, nationally and internationally. The study has a designated Twitter account (@Nursing_Rounds) to support dissemination.^21^

## Discussion

The government's initial response to the Francis Inquiry reports that the majority of hospitals have now implemented IR on their wards. However, there is currently no robust research evidence available to support the benefits of IR or promote its widespread adoption across the UK. The nursing and healthcare workforce are a valuable resource, and it is important that their time and effort is employed in the best way possible to meet patient needs. It is particularly important to ensure that the structured procedure of conducting IR does not simply become a ‘tick-box’ exercise for nursing staff that takes up valuable time without leading to benefits for patients. As with all healthcare interventions, it is also important to provide evidence of the effectiveness and cost of IR, particularly given the increased scrutiny placed on NHS care as a result of the Francis Inquiry and the financial pressures the NHS currently faces. Therefore, there is an urgent need to find out what works in IR, for whom and in what circumstances. The findings of this study will provide robust information about what good practice of IR looks like, how it is delivered and the factors that facilitate or hinder implementation. It will also shed light on poor or ineffectual practice and the factors that influence this. This research will provide benefit by enabling trusts to target their effort and resources on supporting good practice (and redirecting resources from aspects of IR that are not useful) and will inform operational guidelines and policies directing the delivery of direct patient care.
